# Hear our voices: The perceptions and experiences of women who are Deaf on gender-based violence

**DOI:** 10.4102/ajod.v13i0.1490

**Published:** 2024-11-15

**Authors:** Ronel Davids, Maria van Staden

**Affiliations:** 1 Department of Social Work, Faculty of Community Health Sciences, University of the Western Cape, Bellville, South Africa; 2 Gender Equity Unit, DVC Research and Innovation, University of the Western Cape, Bellville, South Africa

**Keywords:** gender-based violence (GBV), women who are Deaf, Deaf community, critical disability theory, support

## Abstract

**Background:**

While all women are at risk of gender-based violence (GBV), it is essential to acknowledge that women are not a homogenous group and that women who are Deaf may experience GBV differently. This study aimed to answer the question: What are the perceptions and experiences of GBV among women who are Deaf?

**Objectives:**

The study’s objectives were to explore and describe these women’s understanding of GBV terminology, their perceptions of GBV and challenges regarding support strategies in place to respond to their unique circumstances.

**Method:**

A qualitative study with four workshops was conducted with 60 participants. The data collected were analysed using thematic analysis.

**Results:**

The results yielded three themes that intrinsically spoke to women’s understanding of GBV terminology, perception of GBV and challenges accessing support. The results highlight that women who are Deaf are at a greater risk of GBV. Participants emphasised the importance of exploring the perceptions of GBV among men who are Deaf. Findings also indicated women’s challenges when seeking support.

**Conclusion:**

The findings underscore the necessity of offering specific assistance to Deaf women facing GBV. Based on the study findings, it can be concluded that those providing support in GBV matters should receive specialised training in GBV, including Deaf culture and South African sign language.

**Contribution:**

The study findings contribute to the field of disability and the development of effective GBV strategies and programmes that are inclusive of women who are Deaf within a South African context.

**Of Note** – The article followed the convention of capitalising ‘Deaf’^1^

## Introduction

Gender-based violence (GBV) or violence against women and girls (VAWG) is a pervasive phenomenon, which affects one in three women during their lifecycle (World Health Organization [WHO] 2013). It is a global public health burden and an encroachment on human rights (Guedes et al. 2016). Gender-based violence is therefore a significant global public health challenge that deprives the fundamental human rights and jeopardises the well-being of women and girls (WHO 2013).

South African scholars argue that only one in nine incidences of GBV is reported, and the prevalence is therefore higher than the reported statistics. Women reported 36 597 cases of sexual offences in 2018/2019 (Statistics South Africa 2018). The South African President affirmed that there were nearly 73 000 assault cases between July and September 2021, and more than 13 000 of these were domestic violence-related. The South African government subsequently instituted progressive legislation and various GBV policies. The *Domestic Violence Act (1998:1)* explicitly emphasises GBV as a response to the high prevalence of this phenomenon and empathetically refers to the victims of domestic violence as ‘among the most vulnerable members of society’. This legislation therefore acknowledged the severity and social impact of this form of abuse in the South African context.

### Gender-based violence and women with disabilities

Research has emphasised GBV and women for years, but there is limited research on the abuse of women with disabilities (Meyer et al. 2022). Women with disabilities are more vulnerable and susceptible to abuse (Humphrey 2016; Zamora et al. 2023) than women without disabilities (Puri, Misra & Hawkes 2015). Disability is a risk factor for abuse in women, and it influences the dynamics and patterns of violence against women (Namatovu, Preet & Goicolea 2018). Most women with disabilities do not report abuse because they are emotionally, financially, psychologically and physically dependent on others. This dependency increases their risk of abuse, oppression and exploitation. Devandas-Aguilar (2017) highlights that women, young girls and girls with disabilities are overly affected by various forms of GBV, including physical, psychological, and sexual abuse, harassment, coercion, deprivation of liberty, confinement, infanticide, human trafficking, neglect, domestic violence and harmful practices such as forced marriage, female genital mutilation, forced sterilisation and irreversible treatments. This study focusses, however, not on disability and GBV in general but rather on women who are Deaf in the context of GBV.

### Gender-based violence and women who are Deaf

It is essential to acknowledge that women are not a homogenous group. Furthermore, all women are at risk of experiencing GBV but women who are Deaf may experience the phenomenon differently. They are also more susceptible to GBV because of their deafness. Deafness can affect the well-being of women who are Deaf on various levels and deprive them of their quality of life. Deafness influences their access to communication and information negatively and thus affects their social interaction. Deafness can influence their emotional well-being and contribute to isolation, loneliness, anxiety and depression (Haile 2021). There is, however, limited literature on deafness and GBV despite the emotional impact, a lack of communication and information. It is therefore difficult to examine and provide effective intervention to women who are Deaf in the context of GBV (Terlektsi et al. 2019).

Research conducted by Pollard, Sutter and Cerulli (2013) as well as Meyer et al. (2022) found that the prevalence rate of sexual violence against women who are Deaf is double in comparison with their hearing counterparts. A study by Napier et al. (2024) with Deaf female employees in the United Kingdom (UK) reported that their gender and inability to hear caused an unsafe working environment because the employees had difficulty to detect potential threats and communicate their concerns effectively. One of the limited African studies by Green and Nnatuanya (2024) focussed on the lack of awareness about GBV among women who are Deaf, and the inadequate institutional responses and obstacles when they require assistance and access support services. Further studies elucidated that women who are Deaf have a significant higher risk to psychological abuse and physical violence from their partners in comparison with women who can hear (Mastrocinque et al. 2020; Schröttle & Glammeier 2013).

A study by Anderson and Kobek Pezzarossi (2014) wanted to ascertain the explanation of ‘abusive relationships’ by female college students who are Deaf, and they found that these students did not perceive the relationships as ‘abusive’ during physical or sexual assault. These authors argue that these students did not have sufficient knowledge on abuse and the subsequent dangers which could account for the high numbers of abuse and victimisation within the Deaf community. These authors further underscored that women who are Deaf may not only have limited information on the nature of GBV but also on the appropriateness of such violence and ways to address it. This may, therefore, contribute to an increase in violence in women and girls with deafness (Anderson & Kobek Pezzarossi 2014) and a high prevalence of psychological, physical and sexual intimate partner abuse (Pollard et al. 2013).

It is evident from the literature that there is a correlation between various political, cultural, historical and social intersections and disabilities, and deafness (Meekosha & Shuttleworth 2009; Vehmas & Watson 2014). This study was, therefore, based on Critical Disability Theory as the conceptual framework because this theory focusses on external influences such as socialisation and interrogates social norms instead of disability (Schalk 2017). Male dominance and patriarchy are social norms related to GBV, and the study was therefore also based on feminism, which confronts male dominance and the effects of patriarchy on women’s lives. It was important for this study to recognise the influence of patriarchy on the realities of women who are Deaf, specifically in the context of GBV.

### Gender-based violence and service provision to women who are Deaf

Women who are Deaf are predisposed to GBV because of service provision barriers, such as medical and legal information and services (Mastrocinque et al. 2020). Mastrocinque et al. (2020) argue that the lack of information and access to GBV services contribute to GBV in women who are Deaf. Victims of GBV require specialised services, but these services focus mainly on women in general and do not concentrate on the needs of GBV victims who are Deaf. Service providers often have limited essential skills to effectively support victims who are Deaf (Cerulli et al. 2015). Service providers have limited understanding of GBV in women who are Deaf because of limited specialised learning opportunities and insufficient access to health education programmes in sign language (Mckee 2009). Many women who are Deaf are therefore unable to access information, ignore or are unaware of the fact that they are victims of GBV and do not recognise that the violence inflicted upon them is a crime (Mancera et al. n.d.). Mancera et al. (n.d.) argue that women who are Deaf experience not only GBV but also trauma because of the inaccessibility to health and support services. This is distinctly evident in the South African context where there are limited programmes on the specific needs of women who are Deaf and these programmes rarely use South African Sign Language (SASL). It is, therefore, evident that support for women who are Deaf is a particular concern irrespective of the tremendous work from civil society to eradicate GBV and support those affected by this phenomenon.

### Women who are Deaf and gender-based violence terminology

The inability to understand the terminology or vocabulary related to GBV further exacerbates the experiences of GBV by women who are Deaf (Davids 2023). A Deaf person may not understand concepts such as femicide, intimate partner violence, intimidation, force and gender if there is no appropriate sign language to illustrate those concepts. Communication with victims who are Deaf encompasses usually lip-reading (Barnett 2002) and/or writing back and forth notes, and the rest being educated guessing which (Abou-Abdallah & Lamyman 2021) may contribute to ineffective communication, confusion and frustration when they do not understand the words. It is more concerning because women who are Deaf are unfamiliar with GBV terminology and may therefore be unable to communicate their experiences. They understand GBV only on account of their unique lived experiences and perceptions. The lack of understanding of GBV terminology can additionally contribute to frustrations and maladaptive behaviours that compound the recovery from the abuse. Research by Haricharan et al. (2012) focussed on equal access to health services for the Deaf in South Africa. These authors found that women who are Deaf encounter challenges with language, which resulted in misunderstandings and an increased risk to human immunodeficiency virus (HIV). It is, therefore, reasonable to suggest that they may also experience challenges to comprehend the concept of GBV as a result of their limited familiarity with GBV terminology (Mckee 2009).

Women who are Deaf using SASL as their primary means of communication may encounter secondary victimisation in search of assistance with GBV. South Africa’s official recognition of SASL acknowledged the fundamental linguistic human rights of the Deaf community. This recognition necessitates support services in SASL to ensure the complete integration and active participation of the Deaf community within society. A study conducted by Masuku, Moroe and Van der Merwe (2021) on the experiences of women who are Deaf and hard of hearing to access healthcare services in Johannesburg, South Africa, confirmed these women experience alienation, exclusion, marginalisation, discrimination, invisibility, a lack of independence and autonomy. These authors recommended training programmes to address communication, reasonable accommodation and attitudes of healthcare professionals. Our study similarly advocates for the inclusion of SASL in these training programmes.

It is evident from the aforementioned limited studies that there is a noticeable dearth of literature on the lived experiences of women who are Deaf on GBV (Masuku et al. 2021; Napier et al. 2024) and especially in the South African context. These women are largely understudied, and their voices are almost invisible in all areas of scholarship, as stated by Chapple (2019). It is therefore crucial to hear the voices of these women who are Deaf on GBV to provide effective services to support them. This resulted in the following research question for the study: What are the perceptions and experiences of women who are Deaf on GBV? The aim of the study was therefore to determine the perceptions and experiences of women who are Deaf on GBV. The research objectives were to explore and describe the specific understanding of GBV as concept by women who are Deaf; to explore and describe their perceptions and experiences of GBV; to explore and describe the intersections of oppression that inhibit or shape these understandings of GBV, as well as their challenges with support services to GBV in their unique circumstances.

## Research methods and design

### Study approach and design

The study used a qualitative research approach with explorative and descriptive research designs to obtain an in-depth understanding on GBV from women who are Deaf. It was evident from the previous literature that GBV in the context of women who are Deaf differs from women with hearing, and it was therefore imperative to understand their experiences and perceptions of the phenomenon. There are also various political, cultural, historical and social intersections which influence GBV and the subsequent experiences and perceptions of women who are Deaf. Qualitative research was appropriate for this study because it focusses on the experiences of people in their natural environment as well as their functioning and interactions (Theherani et al. 2015).

### Research setting

The study was carried out at three local organisations catering for the Deaf and at one organisation for people with disabilities. The first two sites were a local organisation called the Deaf Community of Cape Town and the Deaf Federation of South Africa, which provides social services to people who are Deaf. The participants were recruited from the organisation’s monthly women’s group programmes with the assistance of the organisation’s directors. The third organisation, called eDeaf, is a skills development training provider that strives to improve the social and economic lives of Deaf persons through empowerment and skills development programmes. The participants were recruited from the skills development programme with the assistance of the development programme manager. The fourth workshop took place at the Orion organisation in Atlantis. These participants were recruited on behalf of the researchers by a community activist who works in the Deaf community in Atlantis and who also secured the venue at the Orion organisation. None of the participants were coerced to participate. Participants had the right to withdraw from the study at any point with the assurance that their data would not be used.

### Study participants and sampling strategy

The study population was defined as all women who are Deaf. The study used purposive sampling and recruited 60 females, and the main inclusion criteria were that the women had to be Deaf, were users of SASL, and were above the age of 18 years. Participants did not need to be married or in a relationship.

### Data collection

The data collection encompassed four workshops each attended by 15–20 women who are Deaf. Workshops are a valuable platform to recognise and investigate complicated issues (Ørngreen & Levinsen 2017). It is a ‘research design and analysis, located within an understanding of learning as situational and contextual … and give participants’ ability to act’ (Ørngreen & Levinsen 2017:70). The use of workshops was consistent with the conceptual framework, Critical Disability Theory, which focusses on the situational and contextual experiences and knowledge of participants instead of disability during GBV. During the workshops, the researchers used a semi-structured interview guide to ask questions. To ensure understanding, the researchers presented the questions and various GBV terms on PowerPoint slides. In addition, the South African Sign Language Interpreter (SASLI) interpreted the questions to the participants to ensure full understanding. Participants answers were video recorded with the participants’ permission. The researchers also ensured open and trusting environments during the workshops where participants could share their experiences.

### Data analysis

Data analysis was based on thematic data analysis methodology by Braun and Clarke (2006). The researchers transcribed the video-taped recordings from the workshops to get an in-depth sense of the data in relation to the research questions, aim and objectives. The researchers included the following six steps during data analysis:

1. familiarised themselves with the data;
2. generated codes from the collected data;
3. identified themes;
4. reviewed the themes;
5. defined and named themes; and
6. produced a report.

The researchers used deductive reasoning in the identification of the themes according to the interview schedule because the researchers deemed it necessary to focus on the literature and Critical Disability Theory and Feminism to provide context specific findings on GBV in women who are Deaf. The principal researcher (a SASL user) also ensured accurate interpretations by the Sign Language Interpreter (SLI) by comparing those with the recorded videotapes. This process contributed to the trustworthiness of the data.

### Ethical considerations

Through a South African Sign Language Interpreter (SASLI), the researchers made sure to obtain informed consent for voluntary participation, which involved getting permission to video record the workshop sessions, ensuring confidentiality and privacy such as maintaining the anonymity of participants’ details and narratives (Strydom & Roestenburg 2022), and minimising the risk of harm to the participants. A female Sign Language Interpreter (SLI) for the workshops was recruited based on her skills, professional conduct, years of experience and good standing in the Deaf community and acceptance by the participants participating in the workshops. The SLI had to sign a confidential form.

Ethical clearance to conduct this study was obtained from the University of the Western Cape, Humanities and Social Science Research Ethics Committee (No. HS22/5/11).

## Results

The data analysis resulted in three themes, which encompassed: (1) a limited understanding of GBV terminology; (2) perceptions of GBV; and (3) challenges and a lack of empathy regarding access to support services.

## Discussion

### Theme 1: Limited understanding of Gender-based violence terminology

Theme one focusses on the understanding of GBV terminology by women who are Deaf. This may give rise to misconceptions and misunderstandings on the characteristics of GBV. The participants narrated how words – spoken words – propagated possible misunderstandings. The following narratives highlighted this issue:

‘[*C*]ertain words used by the hearing people like perpetrator or sexual harassment are words we don’t understand.’ (Participant 4, 45 years old, female)

‘[*T*]he words victim or survivor, what one is better to use or what does it mean to be a survivor?’ (Participant 34, 37 years old, female)

‘I don’t know what they mean by force or control, for me, it means the same.’ (Participant 20, 28 years old, female)

‘What does intimate partner violence mean?’ (Participant 17, 41 years old, female)

‘[*D*]on’t know the word assault, we also don’t know how to sign that word, it must be explained to us to understand.’ (Participant 48, 50 years old, female)

One participant stated:

‘We Deaf people know the sign, but we don’t understand what the word means e.g. physical abuse, I know how to sign it, but I don’t know what it means when they say the word to me.’ (Participant 33, 44 years old, female)

Our findings resonate with the studies by Anderson and Kobek Pezzarossi (2012) and Mastrocinque et al. (2022) in the United States (US), and Napier et al. (2024) in the UK, which affirmed that women who are Deaf often do not necessarily understand the meaning of GBV. A study by Anderson and Pezzarossi’s (2012) among women who are Deaf at a US college campus found that limited knowledge of GBV terminology significantly contributed to a higher prevalence of abuse and victimisation within the Deaf community. This is particularly pertinent given that individuals affected by such experiences may not recognise the warning signs of GBV. Crowe (2017) gives compelling reasons for this phenomenon by stating that the inability to hear and understand GBV terminology contributes to vulnerability and a higher risk of GBV in women who are Deaf. The researchers interrogated these findings by asking the questions: Could these compelling reasons also relate to underreporting by these women of the abuse? Furthermore, could their understanding of GBV terminology allow them to think that such abuse is acceptable? It will be necessary to do additional research to answer these questions among women who are Deaf in the South African context.

### Theme 2: Perceptions on gender-based violence

#### Sub-theme 2.1: The role of the family in perpetuating Gender-based violence

Theme two focusses on the perceptions of GBV by Deaf women. The comments by the participants in sub-theme 2.1 provided valuable insight into these women’s perception of GBV. Their responses indicate that women who are Deaf perceived that other women do not protect them against GBV. It was also evident that the participants experienced power and control by husbands and family in-laws, which was reinforced by culture and religion. This corresponds with the conceptual frameworks of Critical Disability Theory and Feminism which emphasise socialisation, male dominance and patriarchy. The following narratives illustrated the influence of socialisation, social norms and patriarchy on the perceptions of women who are Deaf:

‘I must do as told by the husband’s family; that is abuse.’ (Participant 29, 38 years old, female)

‘Women must obey what they are told by their in-laws – can’t talk back, it’s cultural they say.’ (Participant 16, 27 years old, female)

‘It is your marital duty to your husband; my mother-in-law tells me.’ (Participant 38, 47 years old, female)

‘Mothers-in-law protect their sons – they are aware of the abuse – they allow the abuse to happen to women.’ (Participant 22, 27 years old, female)

‘They (mothers-in-law) protect their sons; they don’t stand up for you; they also abuse us by keeping quiet or forcing us to accept the abuse all in the name of religion.’ (Participant 31, 42 years old, female)

‘I am angry more at his mother – I thought another woman would stand or protect me.’ (Participant 39, 44 years old, female)

The following two comments speak to the feelings to how participants felt:

‘Sometimes you feel powerless and do as they say as you believe them.’ (Participant 27, 28 years old, female)

‘My husband’s mother always protects my husband – I am not important – I am not heard.’ (Participant 42, 55 years old, female)

Zinyemba and Hlongwana (2022) affirm that men are the primary perpetrators of GBV against women. The comments by the participants illustrated that male entitlement and dominance contributed to intimate partner violence, but this was further exacerbated by other women who believe in this entitlement and male dominance over other females. It was evident from the findings that women (mothers-in-law) often contribute towards the perpetuation of male superiority and dominance in the name of culture and/or religion. The women subsequently experienced powerlessness.

Govender (2023) asserts that violent behaviour towards women is entrenched in a patriarchal system and is justified through many cultural and religious beliefs which reinforce power over women. These acts of violence against women are sometimes rooted in male entitlement and control of women and their bodies, with the belief that women must be submissive towards their husbands (Mshweshwe 2020; Sikweyiya et al. 2020). Women who are Deaf experience dual minority statuses because of the intersection of paternalism, which manifests especially in Deaf individuals and patriarchal interpretations on women (Prior 2014). Literature indicated the subjugated experiences of women who are Deaf with their mothers-in-law during GBV. Our results concur with the results of a quantitative study by Filson et al. (2010) on the interrelationship between powerlessness and GBV, which found that women who experienced a sense of powerlessness (as alluded to in the comments) exhibited elevated levels of intimate violence victimisation and increased rates of depression. This can then be related to another force, the phenomenon of ‘hearing-Deaf’ in families, which should be considered in relation to the participant’s comments. The term ‘hearing-Deaf’ can also be referred to as hearing privileges. The comments by the participants illustrated power imbalances between the women who are Deaf and their family in-laws which also mirror the discrepancies between social power and social privilege in hearing privileges. These hearing privileges contribute to the past assertions of Hornung, McCullough and Sugimoto (1981:675), as ‘an increased risk of psychological abuse, an even greater risk of physical aggression, and a still greater increased risk of life-threatening violence’ for women who are Deaf. The study by Anderson and Pezzarossi (2014:413) asserts that ‘the abuse of hearing privilege creates unique relationship dynamics and characteristics that may not be present in other violent relationships’. Therefore, the findings of our study could indicate that hearing privileges differentiate between the experiences of GBV in women who are Deaf and women in general. It is evident from the findings that further research is essential to get an in-depth understanding of the dynamics between hearing and Deaf family members and GBV within these relationships.

#### Sub-theme 2.2: The role of men who are Deaf perpetuating gender-based violence

The role of men is imperative to understand the intersections of oppression that inhibit or shape the understanding of GBV in women who are Deaf. This also concurs with Feminism and Critical Disability theories as conceptual frameworks of the study. The following comments from the participants illustrated that the exploration of men’s understanding of GBV may mitigate the effects of the phenomenon:

‘It would help that Deaf men also understand what GBV is – many of them don’t know what GBV is.’ (Participant 23, 29 years old, female)

‘GBV is not just for us women, they (men who are Deaf) also need to understand how they abuse women, they need to understand GBV words, learn to respect us.’ (Participant 47, 52 years old, female)

‘They (men who are Deaf) need to understand abuse, they need training and need to respect us as women.’ (Participant 19, 31 years old, female)

‘Maybe they don’t understand what is GBV because they think it is normal as they don’t understand.’ (Participant 22, 33 years old, female)

‘GBV is not a women thing alone, the men must also be involved and get this information and what can happen to them if they abuse women.’ (Participant 22, 33 years old, female)

‘They need to know that they can be punished for [*by*] abusing women.’ (Participant 49, 48 years old, female)

These findings illustrated that men should also be included in the understanding of GBV terminology, especially in the context of the Deaf community and sign language. The women emphasised the need to educate men on GBV and the repercussions of the abuse. These comments imply healthy and respectful relationships in which men understand their roles and responsibilities to promote a safe environment. Furthermore, these observations suggest that the participants perceive GBV not solely as a women’s issue but also imply that men who are Deaf should take responsibility for their behaviour and understand the legal implications of GBV. Gumede et al. (2023) emphasise the need for additional research and literature in the sub-Saharan African context on the perceptions and experiences of men regarding GBV. Added to this recommendation, this sub-theme emphasises the need for additional research to understand the perspectives of men who are Deaf on GBV. Such research could contribute to a better understanding of GBV from the perspective of men who are Deaf and, in doing so, modify misconceptions and myths about GBV, which will consequently contribute to more accurate insights and appropriate responses within the Deaf community.

### Theme 3: Challenges accessing support and a lack of empathy

The third theme focusses on the challenges experienced by women who are Deaf to access support services during GBV. Support is intricately linked to the notion of access and is directly associated with attitudes, especially within the context of Critical Disability Theory. The participants reflected on the attitudes of service providers and provided further details on their lived experiences when they searched for support during GBV:

‘I reported my husband to the police for rape – but nothing happened to the case. There was no interpreter for me.’ (Participant 45, 55 years old, female)

‘I don’t know where to go to report GBV or where to learn about GBV.’ (Participant 52, 56 years old, female)

‘They tell us to write because they don’t understand us, but the way we write is not understood by a person who is not Deaf or understands the Deaf.’ (Participant 42, 55 years old, female)

‘I don’t understand the words they used when I went for help.’ (Participant 13, 40 years old, female)

‘I didn’t feel comfortable when telling them what happened, they don’t understand me.’ (Participant 28, 37 years old, female)

‘They (police) laughed at me when I tried to report the case because I was signing.’ (Participant 45, 55 years old, female)

‘They threw the case out of court as there was no interpreter for me.’ (Participant 18, 41 years old, female)

The findings of this study illustrated the communication barriers which underscore the challenges of women who are Deaf during the access to services for GBV. The services are inaccessible because of the communication barriers at police stations and courts, limited access to sign language interpreters and the inability of these women to understand the GBV terminology used by healthcare providers. Participants expressed discomfort in sharing their experiences and were anxious because they could be misunderstood and ridiculed when using sign language or when they have to put their experiences of abuse in writing. They consequently experienced a breakdown in communication. The findings also illustrated the experiences of frustration and vulnerability because a participant could not communicate with the service providers and they did not comprehend her situation. One participant shared her experience of being ‘laughed at’ by the police when she attempted to report a case of abuse, which could have exacerbated her vulnerability to safety (Crowe 2017).

Gender-based violence in women who are Deaf is often ignored despite evidence. Furthermore, service providers do not provide efficient services because they are not trained in Deaf culture and SASL. Moreover, they are ignorant and do not recognise the needs of women who are Deaf to support them (Smith & Hope 2015). Our study findings concur with those of Shavers et al. (2005) on community-based intervention with vulnerable women. Their study underscored access to appropriate emotional, psychological, physical, social and economic resources to mitigate the potential adverse effects of GBV. Furthermore, the study findings raise concerns about the role, attitudes and empathy of service providers, especially concerning their ethical obligations and/or principles and professional standards. These attitudes subsequently affect the quality of support and care received by women who are Deaf. The above-stated remarks underscore the direct impact of communication challenges on the quality of support services to these women. The participants’ experiences corroborate the assertion by Masuku et al. (2021) that training programmes should prioritise the development of communication skills, advocate for reasonable accommodation and assistance, and rectify the attitudes of healthcare professionals. The quotations also concur with the findings in a study by Napier et al. (2024) on the absence of Deaf awareness among service providers. The findings also substantiate the assertion by Masuku et al. (2021:6) that ‘communication challenges result from deficiencies in sign language skills among healthcare professionals treating Deaf women’.

Our findings emphasised the need to provide specialised support to women who are Deaf in the context of GBV. This will minimise the obstacles in their search for assistance and access to support services and address the ineffective institutional responses.

## Conclusion

The aim of the study was to determine the perceptions and experiences of women who are Deaf on GBV. Literature on the prevalence of deafness and GBV is limited despite the emotional impact, communication barriers and information which complicate the lives of those who are most affected by the phenomenon (Terlektsi et al. 2019). This study is significant because it focussed on the perceptions and lived experiences on GBV by women who are Deaf. The main themes acknowledged GBV from the perspective of women who are Deaf. Theme one focussed on the limited understanding of GBV terminology by women who are Deaf. They subsequently experienced challenges in understanding the characteristics of GBV. Their inability to hear and understand important information contributed to their vulnerability and risks to GBV. Theme two focussed on the perceptions of women who are Deaf on GBV. They accentuated the role of family members in GBV (sub-themes 2.1) and referred to other women (mothers-in-law), which contributed to male superiority and dominance. They also illustrated the privilege of hearing versus deafness in the discrepancies of social power in families. Their narratives underscore the powerful positions of men towards women and the influence of religion and culture on GBV. The women suggested, in sub-theme 2.2, the inclusion of males, especially those who are Deaf, in understanding and being educated on GBV. These males should not only understand the terminology related to GBV in the context of deafness and sign language but also their role and responsibilities to promote a safe environment. Theme three focussed on the challenges experienced by women who are Deaf to access support services during GBV. This theme demonstrated the negative attitudes of service providers, and it was evident that there should be additional training to provide specialised and empathetic support services to women who are Deaf in the context of GBV.

The conclusion of the study was that the voices of women who are Deaf are not heard irrespective of the higher risk to GBV. They have limited support and subsequently experienced oppression from their families, male counterparts as well as social institutions, for example, healthcare and justice. There are persistent high levels of VAWG which result in intensified feelings of insecurity among women. This study focussed on the voices of women who are Deaf in the context of GBV to interrogate the social norms and structures in society as proposed by Critical Disability Theory and Feminism. The research study contributes to the growing body of knowledge on the circumstances and needs of people with disability and in this case, for women who are Deaf to ultimately ensure their inclusion and participation in matters that affect their lives.

## Footnotes

1 **Of Note** – The article followed the convention of capitalising ‘Deaf’ to signify cultural Deaf identity, and those that rely on South African Sign Language (SASL) as their primary mode of communication as opposed to lowercase ‘deaf’ referring to audiological hearing loss.
